# A novel standardized deep sequencing-based assay for hepatitis C virus genotype determination

**DOI:** 10.1038/s41598-018-22614-0

**Published:** 2018-03-08

**Authors:** Christophe Rodriguez, Alexandre Soulier, Vanessa Demontant, Lila Poiteau, Mélanie Mercier-Darty, Magali Bouvier-Alias, Jean-Michel Pawlotsky, Stéphane Chevaliez

**Affiliations:** 1 National Reference Center for Viral Hepatitis B, C and D, Department of Virology, Hôpital Henri Mondor, Université Paris-Est, Créteil, France; 2 INSERM, U955 Créteil, France

## Abstract

Hepatitis C virus (HCV) genotype and subtype (1a/1b) identification is needed to tailor anti-HCV therapy. Currently available methods accurately identify the genotype and differentiate subtypes 1a from 1b. However, these assays have not been designed to identify other HCV subtypes, nor to recognize mixed genotype/subtype infections, emphasizing the need for a high-resolution system based on phylogenetic analysis of reads obtained by deep sequencing of a relevant genome region. The aim of this study was to evaluate the performance of the Sentosa SQ HCV Genotyping Assay, a novel deep sequencing-based assay targeting the HCV nonstructural 5B (NS5B) region, in clinical samples from patients with an indication for anti-HCV therapy. A high concordance rate with Sanger sequencing of the NS5B region, the reference method, was found for genotype 1 to 6 determination, 1a/1b subtype identification, and genotype 4, 5 and 6 subtyping. Discrepancies were seen essentially for HCV genotype 2 subtyping. Overall, the performance of the deep sequencing-based assay in generating the genotypes/subtype information needed to tailor anti-HCV treatment was adequate in this study. Further improvements, such as a longer NS5B fragment analyzed and enriching the database of reference prototype strains used for subtype assignment would make it a method of choice for HCV genotyping and subtyping for future clinical practice and research.

## Introduction

Hepatitis C virus (HCV) infection is a major global public health problem, with an estimated 71 million people chronically infected worldwide^1^. HCV is classified into seven major genotypes (1–7) and 67 subtypes^2^. Recently, an eighth HCV genotype has been identified in patients from Punjab^3^. HCV diversity results from properties of its RNA-dependent RNA polymerase (RdRp) that randomly introduces synonymous and non-synonymous mutations in viral genomes during replication and lacks proofreading activity to correct these errors. Nucleotide substitutions accumulate, sometimes inducing amino acid changes. Selection of mutant viruses through transmission across patient populations led to the diversification of the different HCV genotypes and subtypes, that subsequently spread over the world through various routes of transmission^4^.

The treatment of HCV infection has been revolutionized with the development and approval of potent and well-tolerated direct acting antiviral (DAA) drug combinations. These therapies yield over 95% cure rates after 8 to 24 weeks of administration in most patient populations^5,6^. However, knowing the HCV genotype (including genotype 1 subtype 1a or 1b) remains necessary to tailor the treatment regimen and its duration^7,8^.

HCV genotype determination methods based on the sole analysis of the 5′ noncoding region (5′NCR) fail to correctly identify HCV subtypes 1a and 1b in approximately 30% and 10% of cases, respectively^9^. Thus, currently available genotyping methods based on reverse hybridization or real-time polymerase chain reaction (PCR) target both the 5′NCR and a portion of another genomic region, such as the core- or nonstructural 5B (NS5B)-coding regions. These techniques are simple to use, accurately identify the HCV genotype and differentiate well subtypes 1a and 1b in the majority of cases^9,10^. However, these widely used assays have not been designed to identify HCV subtypes other than 1a and 1b, nor to recognized mixed genotype/subtype infections. To achieve these goals, a high-resolution system based on phylogenetic analysis of reads obtained by deep sequencing of a portion of the NS5B-coding region was reported to be the ideal method^11–14^. However, no standardized HCV genotype/subtype determination assay based on deep sequencing was available thus far, making this technology unavailable for clinical studies and clinical practice.

The Sentosa SQ HCV Genotyping Assay (VELA Diagnostics GmbH, Hamburg, Germany) is a novel deep sequencing-based *in vitro* diagnostic test comprising a customized version of the epMotion 5075 robotic liquid handling system for RNA extraction and sequence library preparation (Sentosa SX101), a customized version of an Ion One Touch device for template preparation including emulsion PCR (Sentosa ST401), the Ion Torrent technology for deep sequencing (Sentosa SQ301), and software for data analysis and reporting (Sentosa Link and Sentosa Reporter, respectively). HCV genotyping/subtyping is based on deep sequencing of a portion of the NS5B gene (nucleotide positions 990–1677 according to the H77-1a prototype strain) and classification by means of nucleotide sequence homology and phylogenetic analysis.

The aim of the present study was to evaluate the clinical performance of the deep sequencing-based Sentosa SQ HCV Genotyping Assay in a population of patients infected with various HCV genotypes and subtypes representative of those undergoing HCV genotype determination in clinical practice.

## Materials and Methods

### Clinical specimens

A total of 99 consecutive serum (n = 87) and plasma (n = 12) samples collected prior to treatment initiation from patients with chronic hepatitis C infected with HCV genotypes 1 to 6 followed in the Departments of Hepatology of the Henri Mondor university hospital and of the Centre Hospitalier Intercommunal de Créteil were studied. The specimens were frozen and stored at −80 °C until testing. Serum or plasma HCV RNA levels were measured by means of a real-time PCR assay (Abbott RealTi*m*e HCV Assay, Abbott Molecular, Des Plaines, IL)^15^. The limit of detection of this assay is 12 IU/mL (1.1 Log IU/mL).

### Standards

A standard panel, the Hepatitis C Virus Genotype EQA panel (HCVGT17), was purchased from Quality Control for Molecular Diagnostics (Glasgow, Scotland, UK). The panel contains 8 frozen plasmas, including 7 that contain different HCV genotypes and one uninfected. Table 1 shows the HCV genotype/subtype and HCV RNA level in each panel member^16^.

**Table 1:** Standard HCV Genotype EQA panel (Quality Control for Molecular Diagnostics, Glasgow, Scotland, UK). NS5B contig characteristics provided by the Quality Control Report and Pathology Report in the Sentosa SQ HCV Genotyping Assay are shown.

Panel member	Genotype/subtype	HCV RNA level (IU/mL)	NS5B Contig characteristics		
			Median Coverage	No of reads	Length
HCVGT17-01	4a	>10,000	1,079	3,631	682
HCVGT17-02	2b	>10,000	3,072	9,473	576
HCVGT17-03	6a	>10,000	4,605	16,670	680
HCVGT17-04	3a	>10,000	11,875	44,296	682
HCVGT17-05	1b	>10,000	13,585	56,598	683
HCVGT17-06	negative	>10,000	—	—	—
HCVGT17-07	1a	>10,000	8,807	34,294	683
HCVGT17-08	5a	>10,000	8,074	29,548	681

### Study design

The HCV genotype and subtype were determined in the 99 clinical specimens and standard panel members by means of our in-house Sanger sequencing technique targeting the NS5B gene followed by phylogenetic analysis, the reference method for HCV genotype determination^17^. The results were compared with those generated by Sentosa SQ HCV Genotyping Assay and another widely used commercial assay based on reverse hybridization, the line probe assay (VERSANT HCV Genotype 2.0 Line Probe Assay, Siemens Healthcare Molecular Diagnostics, Berkeley, California). The study was conducted in accordance with the International Conference on Harmonisation guidelines, applicable regulations, and the principles of the Declaration of Helsinki. All Patients gave written informed consent for the use of leftover samples.

### Sanger sequencing followed by phylogenetic analysis

Briefly, total RNA was extracted from 400 µL of serum or plasma by means of QIAsymphony DSP Virus/Pathogen kit (QIAGEN GmbH, Hilden, Germany), according to the manufacturer’s instructions. The RNA pellet was eluted with 60 µL of RNAse-free water with 0.04% NaN_3_ and stored at −20 °C until analysis. One-step reverse transcriptase (RT)-PCR was performed with 15 µL of total extracted RNA using the QIAGEN OneStep RT-PCR kit, according to the manufacturer’s instructions. A nested PCR technique was used to amplify an NS5B-coding DNA fragment. The first round used external sense and antisense primers Sn755 and Asn1121^18^ and consisted of 40 cycles at 95 °C for 30 s, 58 °C for 30 s, and 72 °C for 1 min. The second round used internal sense primer NS5B-SI766 and antisense primer NS5B-ASI1110^19^ and consisted of 35 cycles at 95 °C for 30 s, 58 °C for 30 s, and 68 °C for 1 min. PCR products were purified by means of NucleoFast 96 PCR plate kit (Macherey-Nagel GmbH & Co. KG, Düren, Germany) and directly sequenced by means of the BigDye Terminator Cycle v3.1 sequencing kit (ThermoFisher Scientific, Courtabœuf, France) on an ABI 3100 sequencer (Applied Biosystems, Foster City, California), according to the manufacturer’s instructions. Phylogenetic analysis was carried out using genotypes 1 to 7 reference sequences available in GenBank, by means of the Phylogeny Inference Package (PHYLIP), version 3.695^20^. Nucleotide sequences (nucleotide position 724–1009 according to the H77-1a prototype strain) were aligned with the reference sequences using CLUSTAL W^21^. Phylogenetic relationships were deduced by means of DNADIST-NEIGHBOR from PHYLIP. For neighbor-joining analysis, a Kimura 2-parameter distance matrix with a transition/transversion ratio (Ts/Tv) of 2.0 was used^22^. Phylogenetic trees were plotted with FigTree v1.4.3^23^. Their robustness was assessed by bootstrap analysis of 1,000 replicates by means of the SEQBOOT program from PHYLIP. For recombinant 2k/1b strains, the HCV E1 region was amplified and sequenced in addition to the NS5B region, as already described^17^.

### Deep sequencing using Sentosa SQ HCV Genotyping Assay

Briefly, nucleic acid extraction was performed from 530 µL of serum on the Sentosa SX101 robotic instrument using Sentosa Virus Total Nucleic Acid Plus II kit. The NS3, NS5A and NS5B coding regions were RT-PCR amplified by means of Veriti Dx 96-Well Thermal Cycler (Applied Biosystems). After purification of PCR products via magnetic beads, a 200-nucleotide fragment library was prepared on Sentosa SX101. The samples were barcoded by ligation, pooled into a single tube and amplified by emulsion PCR on Sentosa ST401i. Deep sequencing was performed by means of the Sentosa SQ Sequencing Kit on the Sentosa SQ301 Sequencer, based on Ion Torrent technology. Primary data analysis was automatically performed using Sentosa SQ Reporter software. Assembled NS5B contigs (a 685-base pair fragment) were aligned to all NS5B reference sequences using Basic Local Alignment Search Tool (BLAST) and phylogenetic analysis was automatically performed by the software. Based on the manufacturer’s claim, the minimum amount of HCV RNA needed for HCV genotype/subtype determination in the assay is 1,000 IU/mL for genotypes 1a, 1b, 2, 3 and 4 and 2,000 IU/mL for genotypes 5 and 6. For subtypes 1a and 1b differentiation, a 944-base pair fragment in the NS3 region and a 604-base pair fragment in the NS5A region were also sequenced and analyzed by the system. Subtype 1a and 1b sequences were aligned and analyzed through a similar process.

### Reverse hybridization using VERSANT HCV Genotype 2.0 Line Probe Assay

The assay is provided with reagents for PCR amplification of two fragments spanning two thirds of the 5′NCR and a portion of the core-coding region, respectively. After denaturation, the biotinylated PCR products were hybridized to oligonucleotide probes bound to nitrocellulose strips. Each strip carries two control lines, 19 5′NCR DNA probe lines specific for the different HCV genotypes and subtypes, a core control line, and 3 core DNA probe lines that differentiate HCV subtypes 1a and 1b and genotype 6 (subtypes c-l). After hybridization, the non-hybridized PCR products were washed and alkaline phosphatase-labeled streptavidin (conjugate) was bound to the biotinylated hybrid. 5-bromo-4-chloro-3-indolylphosphate (BCIP)-nitroblue tetrazolium chromogen (substrate) reacted with the streptavidin-alkaline phosphate complex, forming a purple-brown precipitate, resulting in a visible banding pattern on the strip. The AutoLiPA 2.0 device (Siemens Healthcare Molecular Diagnostics) was used to carry out hybridizations and the developing color step.

### GenBank/ENA/DDBJ Accession numbers

The accession numbers of the sequences reported in this paper are PRJNA397404.

## Results

### Analytical reactivity of deep sequencing-based Sentosa SQ HCV Genotyping Assay in a standard panel

The HCVGT17 standard panel described in Table 1, representative of HCV genotypes and subtypes commonly found in Europe, was tested with Sentosa SQ HCV Genotyping Assay. The result of the deep sequencing assay was identical to the expected one in the 8 panel members.

### HCV genotyping and subtyping of clinical samples by Sanger sequencing

**Figure 1:**
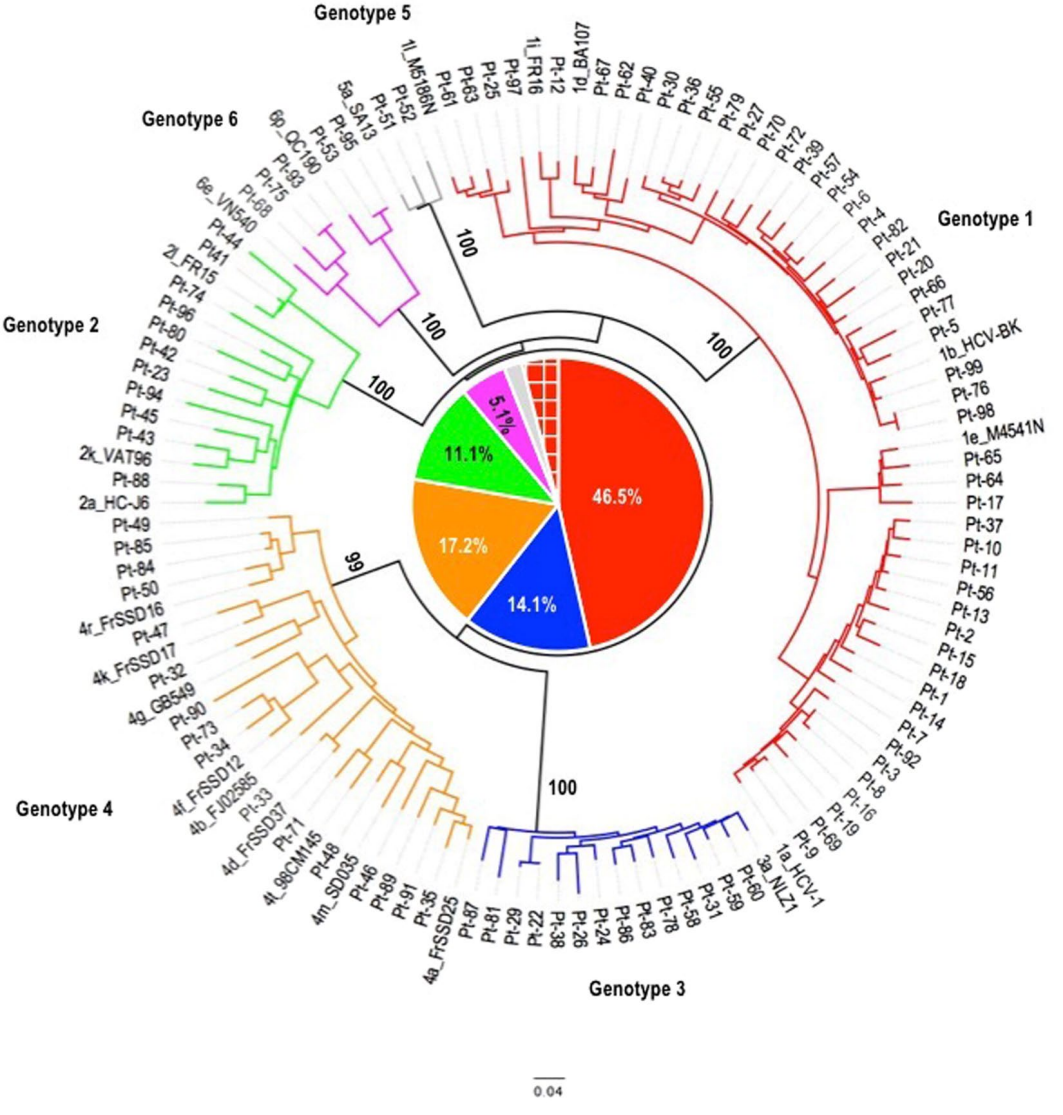
Phylogenetic analysis of HCV NS5B sequences from the 99 patients included in the study. The neighbor-joining tree was based on alignment of nucleotide sequences from a portion of the NS5B gene spanning nucleotide positions 8325 to 9610. Branch lengths were calculated using a Kimura 2-parameter distance matrix with a Ts/Tv ratio of 2.0. Numbers at nodes correspond to the percentage of 1,000 replicates supporting the distal cluster. All reference strains are represented as subtype_isolate ID. In the center of the tree, a pie chart shows the proportions of the different genotypes among the 99 isolates, represented by different colors: red for genotype 1 (46.5%), blue for genotype 3 (14.1%), orange for genotype 4 (17.2%), green for genotype 2 (11.1%), gray for genotype 5 (2.0%), pink for genotype 6 (5.1%) and 2k/1b recombinant strains in hatched red (4.0%).

Our in-house Sanger sequencing method of an NS5B fragment followed by phylogenetic analysis, the standard method for HCV genotyping and subtyping, was used. Among the 99 patients, 46 (46.5%) were infected with HCV genotype 1, 11 (11.1%) with genotype 2, 14 (14.1%) with genotype 3, 17 (17.2%) with genotype 4, 2 (2.0%) with genotype 5, and 5 (5.1%) with genotype 6. The remaining 4 patients (4.0%) were infected with a 2k/1b recombinant strain^24^ (Fig. 1 and Table 2). Table 2 shows the subtype distribution within each genotype category. The subtype was indeterminate in 9 patients (9.1%), including 1 patient infected with genotype 1, 7 with genotype 2 and 1 with genotype 4 (Table 2).

**Table 2:** HCV genotype and subtype in the 99 HCV-infected patients tested using in-house Sanger sequencing followed by phylogenetic analysis of an NS5B fragment, the reference method.

HCV genotype	N (%)	HCV subtype	n
Genotype 1	46 (46.5)	1a	18
		1b	17
		1d	3
		1e	3
		1i	1
		1 l	3
		1 indeterminate	1
Genotype 2	11 (11.1)	2a	1
		2k	1
		2l	2
		2 indeterminate	7
Genotype 3	14 (14.1)	3a	14
Genotype 4	17 (17.2)	4a	4
		4b	1
		4d	1
		4f	2
		4 g	1
		4k	1
		4 m	1
		4r	4
		4t	1
		4 indeterminate	1
Genotype 5	2 (2.0)	5a	2
Genotype 6	5 (5.1)	6e	3
		6p	2
Recombinant 2k/1b	4 (4.0)	2k/1b	4

### HCV genotype and subtype determination with Sentosa SQ HCV Genotyping Assay and VERSANT HCV Genotype 2.0 line probe assay

Table 3 shows the proportion of HCV genotypes and subtypes that were correctly identified by the two commercial assays tested in the study, including the deep sequencing-based Sentosa SQ HCV Genotyping Assay and the line probe assay VERSANT HCV Genotype 2.0. As shown in Table 3, concordance with the reference method was 99–100% at the genotype level. In contrast, concordance at the subtype level was 79.8% with Sentosa SQ HCV Genotyping Assay and 73.7% with VERSANT HCV Genotype 2.0 LiPA (Table 3). The result was not interpretable with Sentosa SQ HCV Genotyping Assay in one case.

**Table 3:** Concordance for HCV genotype and subtype determination of Sentosa SQ HCV Genotyping Assay and VERSANT HCV Genotype 2.0 LiPA with the reference method, in-house Sanger sequencing of the NS5B region followed by phylogenetic analysis.

HCV genotype (reference method^a^)	Patients, n	HCV genotype				HCV subtype			
		Sentosa SQ HCV Genotyping Assay		VERSANT HCV Genotype 2.0 LiPA		Sentosa SQ HCV Genotyping Assay		VERSANT HCV Genotype 2.0 LiPA	
		Concordant n (%)	Discordant n (%)	Concordant n (%)	Discordant n (%)	Concordant n (%)	Discordant n (%)	Concordant n (%)	Discordant n (%)
1	46	45 (97.8%)	1 (2.2%)	46 (100%)	0 (0%)	34 (73.9%)	12 (24.1%)	39 (84.8%)	7 (15.2%)
2	11	11 (100%)	0 (0%)	11 (100%)	0 (0%)	4 (36.4%)	7 (63.6%)	8 (72.7%)	3 (27.3%)
3	14	14 (100%)	0 (0%)	14 (100%)	0 (0%)	14 (100%)	0 (0%)	13 (92.3%)	1 (7.7%)
4	17	17 (100%)	0 (0%)	17 (100%)	0 (0%)	16 (94.1%)	1 (5.9%)	8 (47.1%)	9 (52.9%)
5	2	2 (100%)	0 (0%)	2 (100%)	0 (0%)	2 (100%)	0 (0%)	2 (100%)	0 (0%)
6	5	5 (100%)	0 (0%)	5 (100%)	0 (0%)	5 (1000%)	0 (0%)	3 (60%)	2 (40%)
2k/1b*	4	4 (100%)	0 (0%)	4 (100%)	0 (0%)	4 (100%)	0 (0%)	0 (0%)	4 (100%)
Total	99	98 (99.0%)	1 (1.0%)	99 (100%)	0 (0%)	79 (79.8%)	20 (20.2%)	73 (73.7%)	26 (26.3%)

^a^In addition to Sanger sequencing of the NS5B region, E1 region sequencing was used in 4 patients infected with a 2k/1b recombinant strain.

^*^These strains were identified as recombinant due to the discrepancy between their initial assignment based on the Lipa assay and NS5B sequencing. Recombination was confirmed by sequence analysis of the E1 region spanning the recombining region. Sentosa SQ HCV Genotyping Assay correctly identified genotype 1b in the NS5B region. VERSANT HCV Genotype 2.0 LiPA correctly identified HCV genotype 2 by means of its 5’NCR probes but failed to correctly identify subtype 2k.

### HCV subtyping with Sentosa SQ HCV Genotyping Assay

Sentosa SQ HCV Genotyping Assay correctly identified 73.9% of HCV genotype 1 subtypes (including 94.4% of subtype 1a and 100% of subtype 1b samples), 36.4% of genotype 2 subtypes, 100% of genotype 3a, 94.1% of genotype 4 subtypes, 100% of genotype 5a and all genotype 6 subtypes and the four recombinant strains 2k/1b that were classified as subtype 1b. Among the 20 samples that was not correctly subtyped, the subtype was erroneous in 19 cases and the result was not interpretable in one case (Table 4). In all but one cases, HCV RNA levels measured by the Abbott RealTi*m*e HCV Assay were above 10,000 IU/mL, making it unlikely that these discrepancies were due to the amount of HCV RNA. No other parameter, including the coverage, number or length of reads explained the discrepancies (Table 4). The additional use of NS3 region sequence data correctly identified 88.9% and 82.4% of HCV subtypes 1a and 1b, respectively, while 100% of HCV subtypes 1a and 1b were correctly identified when NS5A region sequence data were used.

**Table 4:** Results provided by Sentosa SQ HCV Genotyping Assay in samples that were incorrectly or not classified, as compared to the reference method based on Sanger sequencing of the NS5B region followed by phylogenetic analysis. NS5B contig characteristics provided by the Quality Control Report and Pathology Report in the Sentosa SQ HCV Genotyping Assay are indicated.

Patient number	HCV genotype and subtype with the reference method	HCV RNA level (Log IU/mL)	HCV genotype and subtype using the Sentosa SQ HCV Genotyping Assay	NS5B contig characteristics		
				Median coverage	No of reads	Length
Pt-25	1l	6.20	1a	16,992	84,058	683
Pt-40	1d	5.90	1b	9,585	37,291	683
Pt-61	1l	5.90	1a	27,221	102,007	683
Pt-62	1d	6.60	1b	11,791	43,735	683
Pt-63	1l	5.00	1b	13,186	56,316	682
Pt-64	1e	5.30	1g	25,458	99,559	683
Pt-65	1e	5.80	1g	23,395	87,406	683
Pt-67	1d	5.90	1b	21,565	71,120	652
Pt-17	1e	6.10	1g	5,864	23,755	681
Pt-12	1i	5.40	1b	21,099	86,173	687
Pt-92	1a	2.70	Not interpretable	1,191	4,473	592
Pt-97	1	5.60	1b	10,454	40,612	677
Pt-41	2l	5.20	2c	15,071	69,466	683
Pt-42	2	4.90	2c, 2m	12,762	47,402	683
Pt-44	2l	6.60	2c	6,189	26,579	681
Pt-45	2	5.50	2a, 2m	15,325	51,871	680
Pt-74	2	6.30	2b	12,559	55,110	684
Pt-80	2	7.00	2c	7,996	31,547	682
Pt-94	2	5.40	2d, 2j	13,342	49,111	683
Pt-46	4	5.00	4a, 4c	11,472	43,868	682

### HCV subtyping with VERSANT HCV Genotype 2.0 line probe assay

The line probe assay failed to correctly identify the HCV subtype in 15.2% of genotype 1, 27.3% of genotype 2, 7.7% of genotype 3, 52.9% of genotype 4 and 40% of genotype 6 cases (Table 3). Concordance with the Sanger method was lower for HCV genotype 2, 4 and 6 subtypes than for HCV genotype 1 and 3 and 5 subtypes (Table 3). Among the 26 specimens that was not correctly classified, the subtype was erroneous in 10 cases and indeterminate in the remaining 16 cases (Table 5). None of the 2k/1b recombinant strains were correctly subtyped as genotype 2k.

**Table 5:** Results provided by VERSANT HCV Genotype 2.0 LiPA in samples that were incorrectly or not classified, as compared to the reference method based on Sanger sequencing of the NS5B region followed by phylogenetic analysis.

Patient number	HCV genotype and subtype with the reference method	HCV RNA level (Log IU/mL)	HCV genotype and subtype using the VERSANT HCV Genotype 2.0 LiPA
Pt-25	1l	6.20	1b
Pt-61	1l	5.90	1b
Pt-63	1l	5.00	1b
Pt-64	1e	5.30	1
Pt-65	1e	5.80	1
Pt-17	1e	6.10	1b
Pt-12	1i	5.40	1a
Pt-41	2l	5.20	2
Pt-44	2l	6.60	2
Pt-74	2	6.30	2a/2c
Pt-87	3a	6.40	3
Pt-32	4g	5.00	4a/4c/4d
Pt-46	4	5.00	4a/4c/4d
Pt-49	4r	6.30	4
Pt-50	4r	5.80	4
Pt-71	4t	6.60	4a/4c/4d
Pt-84	4r	6.45	4
Pt-85	4r	6.30	4
Pt-89	4a	6.15	4
Pt-91	4a	6.10	4
Pt-93	6e	5.10	6
Pt-95	6p	6.40	6c/l
Pt-66	2k/1b	6.40	2
Pt-76	2k/1b	5.10	2
Pt-98	2k/1b	5.80	2
Pt-99	2k/1b	6.60	2

### Reproducibility of Sentosa SQ HCV Genotyping Assay

To verify the reproducibility of the deep sequencing assay, 25 among the 97 clinical specimens were randomly selected and retested once or twice using Sentosa SQ HCV Genotyping Assay in the same or different runs. They comprised 13 genotype 1 samples (including 7 subtype 1a, 5 subtype 1b and 1 subtype 1e), 2 genotype 2 samples (including 1 subtype 2a and 1 subtype 2 l), 4 genotype 3a samples, 3 genotype 4 samples (including 1 subtype 4b et 2 subtype 4r), 1 genotype 5a sample and 1 recombinant 2k/1b strain. The results were identical to the first determination at both the genotype and subtype levels in all instances. The intra- and inter-run precisions were excellent.

## Discussion

HCV genotype determination and subtype 1a/1b differentiation remain needed in clinical practice to choose the most appropriate treatment regimen and the ideal duration of treatment, and to assess the need for ribavirin^7,8^. The most widely used commercial assay for HCV genotype and subtype determination in clinical practice is the reverse hybridization-based line probe assay that uses oligonucleotide probes targeting both the 5′NCR and core-coding region of the viral genome. This assay is limited in the identification of subtypes other than 1a and 1b, mixed infections, recombinant and/or novel strains^25^. Full-length genome sequencing is an alternative method that provides greater resolution. However, it is cumbersome in clinical settings. A promising alternative for HCV genotyping/subtyping is a high-resolution HCV system based on phylogenetic analysis of reads obtained by deep sequencing of an NS5B-coding gene fragment^11,12^.

Sentosa SQ HCV Genotyping Assay is a new automated deep sequencing-based assay that is standardized and easy-to-use without complex training or specialized skills. In this study, we assessed the ability of Sentosa SQ HCV Genotyping Assay to correctly identify the HCV genotype and subtype in clinical samples from patients with an indication for HCV therapy. Our results show good concordance with Sanger sequencing of the NS5B region, the reference method, for genotype determination, 1a/1b subtyping and genotype 4, 5 and 6 subtyping, in keeping with recently presented results^26^. In contrast, the assay disappointingly misclassified 63.6% of genotype 2 subtypes. This was explained by the lack of matching sequences in the assay software database of prototype reference strains. Indeed, we repeated phylogenetic analysis of consensus sequences using a new in-house database of prototype reference sequences and could remove all discrepancies except 4 (1 in a subtype 1d sample and 3 in samples with indeterminate genotype 2 subtype). There was only one genotyping failure with Sentosa SQ HCV Genotyping Assay and the results were 100% reproducible in our experience. Notably, the percent homology with prototype reference sequences from the assay database is not provided in the report. This crucial information should be added for better interpretation of the results.

Subtypes 1a and 1b represent almost 80% of all HCV infections in industrialized countries. Correct identification of these subtypes is strongly recommended by international societies to optimize DAA-based treatment of chronic HCV infection. Preliminary results suggest that some subtypes of non-1 genotypes, especially 2, 4 and 6, respond less well to DAA-based therapies due to the presence of amino acids that reduce their susceptibility to some DAA classes^27^. Thus, HCV subtype determination may become important in clinical research and practice in the future. Thus, performant assays should become available. In this context, the deep sequencing-based Sentosa SQ HCV Genotyping Assay showed excellent performance in determining the HCV genotype, making it a useful tool to apply current HCV treatment guidelines in practice. In contrast, progress remains to be made for subtype identification, essentially through extensive updating of the database used for sequence comparisons and assignment.

Our study has limitations. Among our 99 patients, the distribution of HCV genotypes did not exactly match the distribution observed in France. Indeed, a larger proportion of genotype non-1 patients were included. However, this represents the current epidemiological trend in the country, with a relative decrease of the proportion of genotype 1 infections and an increase in infections with other genotypes, in particular subtypes of genotypes 2 and 4. Our study included only genotype 3 subtype 3a strains, the most prevalent subtype in France, not necessarily reflecting the genetic diversity of this genotype.

In conclusion, this study evaluating the performance of the new deep sequencing-based Sentosa SQ HCV Genotyping Assay for HCV genotype and subtype determination showed that the assay is easy-to-use accurately identifies the HCV genotype and HCV subtypes 1a and 1b. It can thus be confidently used in the current indications of HCV genotype determination in clinical practice. Discrepancies with the reference method were seen at the subtype level, especially for HCV genotype 2 subtypes. To solve this issue, the Sentosa assay will require technical improvements, including increasing the length of the NS5B fragment analyzed and particularly enriching the database of reference prototype strains used for subtype assignment. With these improvements, deep sequencing-based assays are likely to become the method of choice for HCV subtyping in the future.
